# Künstliche Intelligenz in der Urologie – Chancen und Möglichkeiten

**DOI:** 10.1007/s00120-023-02026-3

**Published:** 2023-02-02

**Authors:** Radu Alexa, Jennifer Kranz, Christoph Kuppe, Sikander Hayat, Marco Hoffmann, Matthias Saar

**Affiliations:** 1 Klinik für Urologie und Kinderurologie, Uniklinik RWTH Aachen, Pauwelsstr. 30, 52074 Aachen, Deutschland; 2 Universitätsklinik und Poliklinik für Urologie, Universitätsklinikum Halle (Saale), Halle (Saale), Deutschland; 3 Institut für Experimentelle Innere Medizin und Systembiologie, Uniklinik RWTH Aachen, Aachen, Deutschland; 4 Klinik für Nieren- und Hochdruckkrankheiten, rheumatologische und immunologische Erkrankungen, Uniklinik RWTH Aachen, Aachen, Deutschland

**Keywords:** Maschinelles Lernen, „Deep learning“, Neuronale Netze, Harnblase, Niere, Prostata, Machine Learning, Deep learning, Neuronal networks, Urinary bladder, Kidney, Prostate

## Abstract

Der Einsatz künstlicher Intelligenz (KI) in der Urologie soll zu einer deutlichen Optimierung und Individualisierung der Diagnostik und Therapie sowie einer Kostenreduktion für das Gesundheitssystem beitragen. Die Einsatzmöglichkeiten und Vorteile der KI im medizinischen Bereich werden oftmals unterschätzt oder nur unvollständig verstanden. Dadurch wird die konzeptuelle Lösung von relevanten medizinischen Problemen mit Hilfe von KI-Anwendungen erschwert. Mit den aktuellen Fortschritten der Computerwissenschaften wurden bereits multiple, teils hochkomplexe nicht medizinische Prozesse automatisiert untersucht und optimiert. Die konstante Entwicklung von KI-Modellen kann bei korrekter Anwendung zu einer effektiveren Bearbeitung und Analyse patientenbezogener Daten und einer dementsprechend optimierten Diagnostik und Therapie urologischer Patientinnen und Patienten führen. In dieser Übersichtsarbeit wird der aktuelle Status zur Anwendung der KI in der Medizin sowie deren Chancen und Möglichkeiten in der Urologie aus einer konzeptuellen Perspektive anhand praktischer Beispiele dargestellt.

## Abstract (en)

The use of artificial intelligence (AI) in urology can contribute to a significant improvement with regard to individualization of diagnostics and therapy as well as healthcare cost reduction. The potential applications and advantages of AI in medicine are often underestimated or incompletely understood. This makes it difficult to conceptually solve relevant medical problems using AI. With current advances in computer science, multiple, highly complex nonmedical processes have already been studied and optimized in an automated fashion. The development of AI models, if applied correctly, can lead to more effective processing and analysis of patient-related data and correspondingly optimized diagnosis and therapy of urological patients. In this review, the current status on the application of AI in medicine and its opportunities and possibilities in urology are presented from a conceptual perspective using practical examples.

Durch den Einsatz in unterschiedlichsten medizinischen Bereichen wird die künstliche Intelligenz (KI) stetig optimiert, was in naher Zukunft zu ihrer breiten Anwendung in nahezu jeder medizinischen Subspezialisierung führen wird. Beispielsweise wurde der KI-basierte, genomische Klassifikator „Decipher“ beim Prostatakarzinom als Prognosemarker validiert. Dieser kann zukünftig eine personalisierte, maßgeschneiderte Therapie ermöglichen, indem beispielsweise bei Patienten mit hohen Decipher-Werten im biochemischen Rezidiv eine frühzeitigere multimodale Therapie eingeleitet werden kann [13].

## Einführung in die KI

Die KI-Entwicklung begann im Jahr 1946, als der Mathematiker Alan Turing den ersten modernen Computer namens „Automatic Computing Engine“ konzipierte [23].

Die Kommunikationsebene dieser Maschinen war unidirektional. Ein Computer konnte nur die vom Anwender angeforderten Aufgaben bearbeiten und berechnen. Durch stetig anwachsende Datenmengen und steigende Speicherkapazitäten entwickelte sich parallel die Notwendigkeit einer automatisierten Bearbeitung und eigenständigen Mustererkennung durch Computer in sog. „Big-data“-Bereichen. Durch die klassische Programmierung waren jedoch die Bearbeitung und Interpretation dieser Daten kaum möglich. Mit der Entwicklung von KI-basierten Modellen entstand aus der klassischen, unidirektionalen Kommunikationsebene zwischen Anwender und Computer eine bidirektionale Kommunikation, sodass neue, nicht vordefinierte Muster in multidimensionalen Daten entdeckt und dem Anwender mitgeteilt werden konnten. Als Beispiel sei hier eine Magnetresonanztomographie (MRT) der Prostata dargestellt: Mit einer Auflösung von 1920 × 1080 enthält ein MRT-Bild der Prostata insgesamt 2.073.600 Pixel. Jeder dieser Punkte hat zudem Lokalisations- und Farbkoordinaten. Mit einer klassischen mathematischen Methode wäre es kaum möglich, in dieser multidimensionalen grafischen Darstellung mehrerer Prostatabilder ein spezifisches, medizinisch relevantes Muster zu erkennen und dieses anschließend in eine mathematische Formel umzuwandeln. Die KI-Modelle ermöglichen jedoch ein spezifisches Muster, z. B. eine suspekte Prostataläsion, in dieser großen Datenbank (MRT-Bilder) zu detektieren. Diese modernen Computerarchitekturen wurden so weiterentwickelt, dass sie den neuronalen Strukturen des menschlichen Gehirns ähneln und so selbstständig spezifische Muster in Daten erkennen können.

## Anwendung der KI in der Urologie

**Figure 1:**
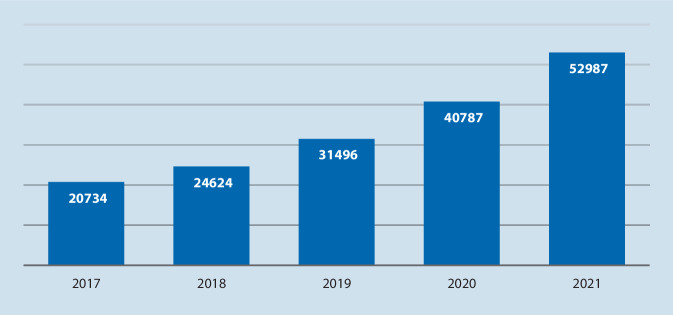

Eine aktuelle PubMed-Suche mit den Schlagworten „prostate cancer“, „artificial intelligence“, „machine learning“ zeigt eine Verdopplung der Publikationen aus diesem Bereich in den letzten Jahren (2018 – 24.624 Publikationen/2021 – 52.987 Publikationen). Der rasante Anstieg KI-assoziierter Publikationen im Bereich der Urologie zeigt eindrucksvoll, dass ein erhebliches Potenzial für computergestützte Untersuchungen vorliegt und dadurch Verbesserungen im Bereich der Diagnostik und Therapie erzielt werden können. (Abb. 1).

## Prostata

Um die Diagnostik des Prostatakarzinoms zu verbessern, wurden verschiedene radiologische Modalitäten erforscht [11]. Während die Computertomographie (CT) aufgrund ihrer eingeschränkten Weichteilcharakterisierung i. Allg. als unzureichend angesehen wird, haben Koreevaar et al. [14] gezeigt, dass eine neue KI-basierte Software für die CT-grafische Diagnostik eines klinisch-signifikanten Prostatakarzinoms geeignet ist. Mit einer 88 %igen Genauigkeit des neuronalen Netzwerks belegt diese Studie, dass CT-Bilder genügend Informationen beinhalten, um klinisch signifikante Prostatakarzinome detektieren zu können [14].

Die bekannte Variabilität des PI-RADS-Scores (Prostate Imaging Reporting & Data System) in Bezug auf die Leseleistung und Erfahrung des befundenden Radiologen kann durch KI-unterstützte Arbeitsabläufe signifikant reduziert werden [24]. Des Weiteren kann eine KI-basierte Software als „concurrent reader“ dem Radiologen als Hilfestellung dienen, exaktere Ergebnisse zu erzielen. So konnte beispielsweise in einer Publikation von Winkel et al. 2021 gezeigt werden, dass für klinisch signifikante Befunde eine Verbesserung der Genauigkeit von 84 auf 88 % durch die KI-Unterstützung erzielt werden kann [24].

Die KI-basierten Werkzeuge kommen auch intraoperativ zur Anwendung. Die automatisierte laparoskopische Kamerapositionierung, die Planung während der automatisierten robotischen Chirurgie zur Vermeidung von Instrumentenkollisionen sowie die Erkennung menschlichen Gewebes zur Vermeidung iatrogener Verletzungen zeigen vielversprechende Ergebnisse. Sie tragen zu einer Reduktion der Operationszeit und damit Steigerung der Kosteneffizienz moderner Medizin bei und steigern die Patientensicherheit im Rahmen verschiedener roboterassistierter Eingriffe [5].

Porpiglia et al. haben ein KI-Modell zur besseren intraoperativen Erkennung einer extrakapsulären Ausdehnung auf das neurovaskuläre Bündel bei der radikalen Prostatovesikulektomie entwickelt [17]. Präoperative MRT-Bilder der Prostata werden während der roboterassistierten Prostatektomie fusioniert und die vom Tumor betroffene Zone der Prostata in einem dreidimensionalen (3D-)Bild dem Operateur aktiv demonstriert. Diese Technik könnte potenziell auch bei der roboterassistierten Nierenteilresektion, insbesondere bei endophytischen oder dorsal gelegenen Tumoren, als Hilfsmittel eingesetzt werden. Diese Art von intraoperativer bildgestützter Navigation kann zur Vermeidung eines positiven chirurgischen Schnittrandes und zur Maximierung eines Organerhalts beitragen.

## Niere

Die bildgebende Unterscheidung zwischen einem benignen Onkozytom und einem Nierenzellkarzinom kann sich aufgrund überlappender Eigenschaften anspruchsvoll gestalten [2]. Vor allem die chromophobe Variante des Nierenzellkarzinoms ist histologisch nur schwierig von einem Onkozytom zu differenzieren [2]. Radiologisch wurde eine Methode entwickelt und validiert, welche diese Aufgabe mit einer Genauigkeit von 100 % meistert [3]. Dafür wird die Kontrastverstärkung im Tumor relativ zu dem angrenzenden Gewebe gemessen. Der Ansatz erfordert eine manuelle Lokalisierung und Messung des Spitzenbereichs der Kontrastverstärkung innerhalb des Tumors. Dadurch wird die Untersuchung subjektiv und prädisponiert zu den damit einhergehenden Einschränkungen. Als automatisierte Lösung dieses Problems haben Baghdadi et al. eine KI-basierte Software mithilfe von CT-Bildern entwickelt, welche mit einer Sensitivität von 100 % und einer Spezifität von 89 % diese zwei Entitäten voneinander unterscheiden kann [3]. Nur ein Tumor von 20 wurde von diesem Modell nicht korrekt eingestuft. Die Tumorgröße war in diesem Fall die kleinste von allen (1,4 cm), was möglicherweise auf eine geringere Empfindlichkeit dieser automatisierten Methode bei kleineren Tumoren schließen lässt. Mit der Erweiterung der Trainingsdaten und nach weiterer Optimierung können solche Modelle als Unterstützung in der radiologischen Diagnostik von Nierentumoren eingesetzt werden.

## Harnblase

Die Weißlichtzystoskopie (WLC) der Harnblase stellt den Standard in der primären Diagnostik des Harnblasenkarzinoms dar. Die Sensitivität und Spezifität der Weißlichtzystoskopie in der Detektion von Harnblasenkarzinomen ist bekanntermaßen limitiert und liegt untersucherabhängig zwischen 62–84 % (Sensitivität) und 43–98 % (Spezifität; [7]).

Zur Steigerung der Detektionsrate wurden in der Vergangenheit neuartige Techniken wie die fluoreszenzassistierte transurethrale Resektion der Harnblase mit Hexaminolevulinat (HAL) bzw. 5‑Aminolävulinsäure (5-ALA; in Europa keine Zulassung für die Verwendung im Harnblasenkarzinom) oder das NBI etabliert [9, 22]. Als einen ersten Schritt in der Automatisierung der zystoskopischen Diagnostik von Urothelkarzinomen der Harnblase wurde von Ali et al. eine KI-basierte Software zur Erkennung und histologischen Vorhersage des Tumorstadiums basierend auf endoskopischen Bildern entwickelt und trainiert [1]. Die Software erreicht eine Sensitivität von 96 % und eine Spezifität von 89 % in der Erkennung von Bildern mit malignen Läsionen. Zusätzlich konnte erstaunlicherweise eine Vorhersage der Tumorinvasion (T1 und T2) – nur auf Bildern basierend – von 100 % bzw. 91 % erzielt werden [1]. Die Ergebnisse weisen auf das Potenzial der neuronalen Netzwerke für die Etablierung von Klassifikations- und Prädiktionsmodellen für die Diagnostik von Blasenkrebs auf Basis zystoskopischer Bilder hin [1].

Entsprechend der pathologischen Diagnose der Harnblasentumoren wurde auch ein KI-Modell in der molekularen Erkennung eingesetzt. Es wurde bereits gezeigt, dass die molekularen Subtypen (luminal, basal-squamös und neuronal) eines muskelinvasiven Harnblasentumors mit der Prognose sowie dem Ansprechen auf eine neoadjuvante Therapie zusammenhängen [19]. Die molekulare Erkennung basiert auf komplexen, zeit- und kostenintensiven Methoden, weswegen Woerl et al. ein neuronales Netzwerk als Lösung dieses Problems untersuchten [25]. Dieses wurde an histopathologischen Präparaten (Hämatoxylin-Eosin-Färbung), ohne weitere immunhistochemische Analyse, zur Erkennung von molekularen Subtypen trainiert und erreichte eine kombinierte Genauigkeit von 89 %. Des Weiteren hat diese KI-basierte Software als „concurrent reader“ die Genauigkeit der Pathologieexperten in der Subtypendifferenzierung von 38,2 auf 58,9 % gesteigert [2]. Nach weiterer Validierung hat diese KI-Anwendung viel Potenzial in der Verbesserung der Detektionsrate von molekularen Subtypen des muskelinvasiven Urothelkarzinoms anhand der standardisierten histopathologischen Präparate ohne den Einsatz von zusätzlichen immunhistochemischen Untersuchungen.

## Molekulare Analyse

Wegen der großen in Proteom‑, Genom- und Transkriptomanalysen enthaltenen Datenmengen werden zur Entdeckung neuer prädiktiver Marker in den letzten Jahren diverse KI-Methoden in der molekularen Analyse eingesetzt.

Um den Dedifferenzierungsstatus der Tumorzellen und dadurch ihre Aggressivität zu objektivieren, wurden mit Hilfe von KI-Methoden in einer Datenbank aus Transkriptom‑, Methylom- und Transkriptionsfaktorbindungsstellen zwei Scores für die epigenetischen Merkmale (mDNAsi) bzw. ihrer Expression (mRNAsi) in Tumorzellen entwickelt [15]. Diese sind in der Entdeckung von neuen urologischen Biomarkern essentiell, da sie ihrerseits bei der Beurteilung der Tumoraggressivität, Evaluierung des Metastasierungsrisikos und Auswahl eines individualisierten Therapieplans sinnvoll eingesetzt werden können.

Analog wurde in einer Datenbank mit genetischen Merkmalen von Prostatakarzinompatienten das Muster von 22 Genen (Decipher) präziser als die herkömmlichen klinisch-pathologischen Merkmale entdeckt. Dieses kann Patienten mit einem Prostatakarzinom anhand der Wahrscheinlichkeit einer Metastasierung in Low‑, Intermediär- und Hoch-Risiko-Gruppen einteilen. Folglich wird eine individualisierte und maßgeschneiderte Therapie ermöglicht [21].

Um die Detektion eines klinisch signifikanten Prostatakarzinoms in biopsienaiven Patienten zu verbessern, wurde anhand von Daten aus extrazellulärer Vesikel-RNA und Metaboliten des Urins das ExoSpec-Model entwickelt. Bestehend aus vier Gentranskripten, sechs Peptiden und zwei klinischen Variablen bietet dieser Marker einen Vorteil gegenüber dem „standard of care“ und könnte unnötige Biopsien um 30 % reduzieren [16].

## Prinzip der KI

Da KI auf Mathematik und Software als Kommunikationsmedium zwischen Menschen und Computer basiert, ist der Unterschied zwischen der klassischen Kommunikationsart und der KI-basierten Art hilfreich für das Verständnis des Sachverhalts.

**Figure 2:**
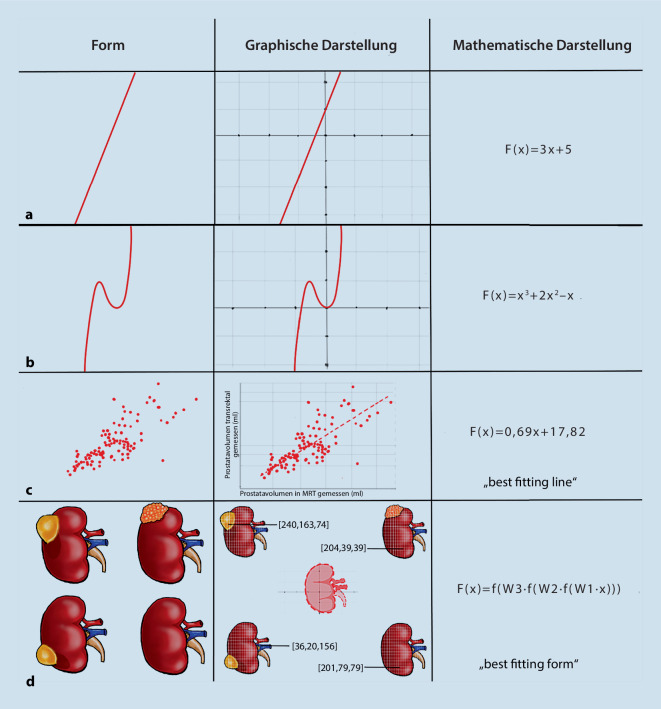

Mittels Programmiersprache (Software) als mathematisches Kommunikationsmedium kann der Mensch dem Computer mitteilen, welchen Prozess die Computerkomponente (Hardware) durchführen muss. Ebenso kann der Computer mittels einer mathematischen Methode (Mustererkennung) übersetzen, wie ein Prozess (Form, spezifische Datenbank) mathematisch konzipiert ist (z. B. mathematische Übersetzung einer Linie entspricht F(x) = 3x + 5, einer kubischen Parabel entspricht F(x) = x^3^ − 2 × ^2^ + x, einer komplexeren Datenbank „best fitting line“ entspricht 0,69x + 17,82; Abb. 2). Der Prozess der Mustererkennung muss vordefiniert sein, also zuerst vom Menschen mathematisch verstanden und anschließend mittels Software dem Computer mitgeteilt werden.

Mit einer komplexen multidimensionalen grafischen Darstellung wird die mathematische Übersetzung komplexer Daten (Bilder, Sprachen und andere Big Data) mit klassischer Mathematik immer schwieriger. Dadurch wird eine Musterentdeckung drastisch erschwert, wenn nicht sogar unmöglich.

## Einsatz neuronaler Netzwerke

Als Problemlösung dient das maschinelle Lernen, welches auf Generierung von Wissen durch Mustererkennung aus Datensetzen basiert und z. T. auf Deep-learning-Algorithmen fußt. Diese sind in der Lage, Daten mit einer neuronalähnlichen logischen Struktur zu analysieren.

Intra- und interneuronale physiologische Funktionen von Neuronen wurden beispielsweise mit der Hoffnung einer automatischen Mustererkennung mathematisch in komplexe Datensets übertragen [20]. Unter *BioMimikry *versteht man somit die Nachahmung natürlicher Erkennungsprozesse durch menschliche Neuronen. Nach mehreren Versuchen, Optimierung und Anpassung künstlicher neuronaler Schichten sowie gleichzeitiger Entwicklung von hochleistungsfähigen Recheneinheiten konnten die Computer in großen Datenmengen neue, nicht vordefinierte Muster erkennen [6]. Dadurch kommt auch der intelligente Charakter der neuronalen Netze zum Ausdruck, der ihnen den Namen „künstliche Intelligenz“ verlieh. Wie in Abb. 2d konzeptuell dargestellt, werden den neuronalen Netzen die initialen Daten (z. B. unterschiedliche Nierenbilder, jeweils aus Millionen Pixeln bestehend) präsentiert, so dass diese ein Muster, passend zu dem korrekten Ergebnis, in unserem Fall ein Nierenprofil finden können („best fitting form“). Ohne die künstlichen neuronalen Netzwerke wäre die Bearbeitung bzw. die Nutzung und die Mustererkennung solcher komplexen Datensets nur äußerst mühsam möglich.

## Vor- und Nachteile der KI

Patientinnen und Patienten suchen bei einer Erkrankung hauptsächlich eine auf Einfühlsamkeit, zwischenmenschliche Fähigkeiten und kompetenzbasierende Interaktion mit einem Mediziner. Im Bereich der „,soft skills“ zeigen die KI-Anwendungen relevante Fortschritte in den letzten Jahren, sind jedoch noch in der Entwicklungsphase. So wurden KI-Modelle bereits in unterschiedlichen Arbeitsgruppen für das Training und die Evaluation von „soft skills“ wie Führung, Teamarbeit, Verhandlung, Empathie und kulturelles Bewusstsein eingesetzt [8]. Derartige KI-Modelle bieten beispielsweise das Potenzial medizinisches Personal in Bezug auf „soft skills“ zu verbessern, um perspektivisch hierdurch ihre eigenen Fertigkeiten und Eigenschaften zu optimieren.

Die Standards zur Bewertung der Qualität, Sicherheit und Verantwortung sind Hauptbestandteil der Forschung von KI-Anwendungen. Diese Charakteristika sind jedoch in vielen Länder noch im Etablierungsprozess [18]. Hacker können Informationen verfälschen, welche möglicherweise schwerwiegende Fehler verursachen können. Ohne Sicherheitsüberprüfungen könnten die Benutzer besorgt sein, dass die KI-basierte Software zu falschen Diagnosen oder falschen Behandlungen führt. Trotz der Nachteile der KI-Anwendungen kann das Gesundheitssystem mit einer frühzeitigen Erkennung und Beseitigung dieser, sowie einem adäquaten Einsatz dieser Technologie von der Stärke der KI profitieren. Mit einer großen Rechenleistung kann KI-basierte Software die Diagnose und Vorhersagefähigkeit von Patientenerkrankungen erheblich verbessern [4]. Durch die hohe Genauigkeit und Geschwindigkeit von automatisierten medizinischen Abläufen kann die KI-basierte Software medizinisches Personal unterstützen. Beispielsweise hat sich gezeigt, dass eine KI-Anwendung in der Lage ist, Hautkrebs effizient zu diagnostizieren und damit dem Dermatologen eine zusätzliche Hilfestellung zu bieten [10]. In Rahmen des Projekts „KI-unterstützte Therapiebegleitung von Tumorpatienten am Beispiel der Urologie“ (KITTU) an der Universitätsmedizin Mainz wird ein KI-Assistenzmodell konzipiert, um die optimale Behandlungsoption bei urologischen Tumorpatienten zu eruieren und die involvierten Ärzte bei der Therapieentscheidung zu unterstützen [12]. Die KI-basierte Analyse von komplexen Patientendaten ermöglicht die Steigerung der Qualität von interdisziplinären Therapieempfehlungen und dadurch die Optimierung der uroonkologischen Behandlung. Dazu wird diese KI-basierte Software den notwendigen Aufwand der administrativen Tätigkeiten im Rahmen des Tumorboards reduzieren [12]. Des Weiteren kann der automatisierte Charakter KI-basierter Anwendungen zu relevanten ökonomischen Vorteilen des Gesundheitssystems führen.

## Fazit für die Praxis

- Mit dem Aufbau des ersten modernen Computers von Alan Turing im Jahr 1946 wurde die Entwicklung der künstlichen Intelligenz initiiert.
- Die Anzahl stetig wachsender Publikationen zum Thema künstliche Intelligenz (KI) in der Urologie zeigt das Potenzial bei der Optimierung von Diagnostik und Therapie auf.
- Durch das Schaffen eines besseren Grundlagenverständnisses kann das Vertrauen in die KI in Bezug auf Forschung und Einsatz in der Patientenbetreuung erhöht werden.
- Durch das frühzeitige Erkennen und die Reduktion von Schwachstellen der aktuell in der Wachstumsphase befindlichen KI-Anwendungen kann medizinisches Personal zukünftig von ihren Stärken profitieren und dadurch die Patientenbetreuung in der Betriebsamkeit des Alltags optimieren.
